# Prevalence of occult hepatitis B infection among treatment-naive persons living with HIV in Ghana

**DOI:** 10.1371/journal.pone.0305862

**Published:** 2024-06-26

**Authors:** Emmanuel Salia, Yvonne Ayerki Nartey, Francis Tanam Djankpa, Faustina Pappoe, Samuel Victor Nuvor, Dorcas Obiri-Yeboah

**Affiliations:** 1 Department of Microbiology and Immunology, School of Medical Sciences, University of Cape Coast, Cape Coast, Ghana; 2 Central Laboratory Sub-BMC, Korle-Bu Teaching Hospital, Accra, Ghana; 3 Departmment of Internal Medicine and Therapeutics, School of Medical Sciences, University of Cape Coast, Cape Coast, Ghana; 4 Department of Internal Medicine, Cape Coast Teaching Hospital, Cape Coast, Ghana; 5 Department of Anatomical Pathology and Physiology, School of Medical Sciences, University of Cape Coast, Cape Coast, Ghana; University of the Witwatersrand, SOUTH AFRICA

## Abstract

Hepatitis B virus (HBV) constitutes a significant global health challenge, with more than 2 billion people infected globally and almost 291 million chronic cases. In Africa, coinfection of HBV with Human Immunodeficiency Virus (HIV) is high, yet the condition remains overlooked in many countries. While antiretroviral therapy (ART) has improved HIV survival, viral hepatitis continues to contribute to morbidity and mortality. Occult Hepatitis B infection (OBI), characterized by a low-level of HBV DNA in individuals with negative hepatitis B surface antigen (HBsAg), is an emerging concern among HIV seropositive individuals due to the risk of HBV reactivation and associated complications, especially hepatocellular carcinoma (HCC). Ghana has an estimated HBV/HIV coinfection prevalence of 13.6% making it important to also determine potential cases of OBI. This study aims to assess OBI prevalence in persons living with HIV (PLHIV). A cross-sectional study was conducted in five health facilities in the Cape Coast Metropolis. HBV-related serological markers were determined among 116 PLHIV using the Enzyme-Linked Immunosorbent Assay (ELISA) method. HBV DNA was extracted from 30 participants found to be HBsAg negative but positive for hepatitis B core antibody (HBcAb+). Nested PCR was employed in detecting HBV DNA and HBV viral load was performed using qPCR. The median age of the participants was 37 years (IQR 22–65). Serologically, 7.8% (n = 9, 95% CI: 3.5–22.7), 12.1% (n = 14), and 25.9% (n = 30) tested positive for solely HBsAg, HBsAb, and HBcAb respectively. OBI prevalence among HBsAg-/HBcAb+ participants was 16.7% (n = 5, 95% CI: 6.5–23.7) with a median HBV DNA level of 139.2 IU/ml (IQR, 96.7–142.0). The prevalence of OBI among HIV-positive participants in the Cape Coast Metropolis highlights the need to consider screening for HBV among HIV patients using nucleic acid amplification tests. This can inform medical management and reduce the risk of liver complications, including HCC.

## Introduction

Hepatitis B virus (HBV) is endemic in parts of Sub-Saharan Africa (SSA), East and Southeast Asia, [1] and is the major cause of acute and chronic liver diseases including liver cirrhosis and hepatocellular carcinoma (HCC) [2,3]. Over two billion persons worldwide have been infected with HBV, [4] and 291 million out of this number are chronically infected [5]. Furthermore, it is estimated that 1.5 million new infections and 820,000 deaths in 2019 were due to HBV infection [6]. The World Health Organization (WHO) has therefore prioritized viral hepatitis elimination in its public health agenda. The Global Health Sector Strategy on viral hepatitis in 2022 proposed strategic changes to eliminate chronic HBV as a global health threat by 2030 through improved testing, treatment access, simplified service delivery, and by addressing challenges faced by at-risk populations [7].

Serological markers, particularly hepatitis B surface antigen (HBsAg), have traditionally been employed for the diagnosis of HBV. Nevertheless, advancements in molecular methodologies have provided supplementary avenues for HBV diagnosis [8]. Molecular testing has enabled the detection of HBV DNA in persons who are HBsAg-negative, a state that defines occult hepatitis B infection (OBI) [9]. OBI is often characterized by the presence of HBV DNA, typically at levels less than 200 IU/mL in the blood and/or liver tissue, along with the absence of serum HBsAg [9]. The absence of HBsAg in the bloodstream is thought to be a result of decreased production or expression of HBsAg as well as a reduced rate of virus replication due to mutation on the “a” determinant [10]. These factors could influence the ability of conventional assays to detect HBsAg [11]. Transmission of OBI has been confirmed through multiple routes, including blood transfusion, organ donation, and vertical transmission [12], leading to chronic HBV infection and associated liver complications [11,12]. Consequently, there is a need to determine OBI burden in endemic regions and in susceptible populations.

The co-infection of Human Immunodeficiency Virus (HIV) and HBV is facilitated by the presence of similar transmission routes and risk factors for both viruses [13]. It is well-established that HIV infection disrupts the entire immune system [14] and enhances hepatic damage via immune activation and inflammation [15]. This creates a microenvironment for HBV persistence in the liver without detectable levels of HBsAg in the blood [16] and contributes significantly to the development of OBI. HIV exerts a significant adverse impact on the natural course of HBV infection. This influence manifests as an acceleration of HBV progression to conditions such as cirrhosis and HCC, along with a higher incidence of reactivation in previously inactive HBV carriers [17,18]. The advent of antiretroviral therapy (ART) has led to substantial improvements in the survival of persons living with HIV (PLHIV) [19]. However, it is imperative to acknowledge that viral hepatitis remains a potential source of morbidity and mortality among PLHIVs if it is not promptly identified and managed [20]. The pooled prevalence of HBV-HIV co-infection in Ghana is 13.6% [21]. Consequently, this study aimed to determine OBI prevalence in PLHIV.

## Materials and methods

### Study setting

A cross-sectional study was conducted from November 2019 to August 2020 at the ART clinics of five health facilities within the Cape Coast Metropolis. These facilities include Cape Coast Teaching Hospital (CCTH), Ewim Urban Health Centre, Cape Coast Metropolitan Hospital, University of Cape Coast Hospital and Elmina Urban Health Centre. The Cape Coast Metropolis covers a land size of 122km^2^ and has a population of 189,925 which include 97,135 females and 92,790 males. The Metropolitan District includes both Cape Coast South and North municipalities. [22]. The CCTH is a 400-bed capacity facility that serves as a referral center for health facilities in the Central, Western and Western North regions respectively. It is the only tertiary hospital on the western coast of Ghana. The Cape Coast Metropolitan hospital is located at Bakano in the Cape Coast south municipality. It is a primary health facility that runs a 24-hour service, providing primary health care to inhabitants in the metropolis and augments the high patient turnouts at the CCTH. The University of Cape Coast Hospital is an 80-bed capacity facility situated in the southern campus of UCC that provides primary health care to the university community and indigenes of the surrounding communities. Ewin and Elmina Urban Health Centers located in Elmina and Cape Coast south municipalities respectively play a vital role in comprehensive healthcare and disease prevention through a 24-hour primary care, preventive care, and community healthcare services to members of their surrounding communities.

### Study population

Adults aged 18 years and above who were diagnosed with HIV infection who had either not started ART or who had defaulted treatment for at least 6 months were recruited from the selected health facilities.

### Ethical consideration and participants’ consent

The study was approved by the University of Cape Coast Institutional Review Board (UCCIRB) with ethical number UCCIRB/EXT/2019/22. In addition, permission was sought from the management of the health facilities where participants were recruited. Written informed consent was obtained from all participants who met the study’s inclusion criteria, and their confidentiality and anonymity were preserved by using unique identification numbers for each participant.

### Sample size

In this study, a total sampling approach was used to recruit all eligible participants who provided informed consent. The COVID-19 pandemic resulted in much lower new OPD clinic attendance including at ART clinics. Consequently, the recruitment period was extended until at least 100 participants could be recruited.

### Inclusion and exclusion criteria

The study included both men and women aged 18 years and older diagnosed with HIV who had either not started ART or had defaulted ART treatment for at least six months. Additionally, participants had to have tested negative for HBsAg. Exclusion included the paediatric population, patients currently on ART or those already known to have HBV infection.

### Data collection and testing

A structured questionnaire was used to obtain demographic information as well as the HBV vaccination history of participants. A volume of 3-4mls of venous blood was obtained from study participants into ethylenediamine tetra acetic acid (EDTA) and gel tubes respectively. Gel tube samples were allowed to clot and centrifuged to separate blood cells from serum. Plasma was aseptically pipetted into sterile labelled cryotubes. Serological testing was performed using conventional enzyme linked immunosorbent assay (ELISA) protocol for the identification and confirmation of HBsAg, HBsAb, HBeAg, HBeAb and HBcAb (IgM/IgG) using BIORAD kits (Monolisa HBsAg ULTRA, Monolisa Anti-HBs PLUS, Monolisa HBeAg ULTRA, Monolisa Anti-HBe PLUS and Monolisa Anti-HBc PLUS, BIORAD, France). DNA extraction for participants with HBsAg-/HBcAb+ infection and pooled samples of participants with HBsAg-/HBcAb- infection was performed with 100ul of plasma using Zymo Research Quick-DNA^TM^ Miniprep Plus kit according to the manufacturer’s protocol. The Zymo Research Laboratory Diagnostic DNA extraction kit protocol uses the principle of DNA binding to silica membranes, selective washing to remove contaminants and elution to recover highly purified DNA. The plasma samples were subjected to the genomic lysis buffer to lyse the cells. After a washing step with DNA pre-wash and g-DNA buffers to rid the sample of contaminants, the nucleic acid was recovered by elusion in a mild alkaline elution buffer. HBV DNA extracts were amplified using nested PCR technology with primers targeting the HbsAg. The initial round of PCR was performed for S-gene using the primers MD14 (nt 418–433, 5’-GCGCTGCAGCTATGCCTCATCTTC-3’) and HCO2 (nt 761–776, 5’-GCGAAGCTTGCTGTACAGACTTGG-3’). The PCR reaction began with a 5-minute hot start, followed by 40 cycles of denaturation at 94°C for 45 seconds, annealing at 55°C for 45 seconds, and extension at 72°C for 60 seconds. Subsequently, the second round of PCR was carried out on 2 μl of the products obtained from the first round in a 25 μl reaction. For the second round, the primers used were ME15 (nt 455–470, 5’-GCGCTGCAGCAAGGTATGTTGCCCG-3’) and HDO3 (nt 734–748, 5’-GCGAAGCTTCATCATCCATATAGC-3’). Similar to the first round, the second round PCR began with a 5-minute hot start, followed by 35 cycles of denaturation at 94°C for 45 seconds, annealing at 55°C for 45 seconds, and extension at 72°C for 60 seconds.

The Applied BioSystems ^TM^ TaqMan® qPCR technology version 2.0 (Roche Diagnostics, Germany) which has a detection limit of 20 IU/mL was used. Primers targeting the HBV core genes were used in estimating HBV viral load of the study samples. OBI was defined by the presence of HBV DNA in HBcAb positive participants only (HBsAg negative, HBsAb negative and HbcAb positive).

### Data analysis

Data was cleaned and exported into Stata version 16 (College Station, TX: Stata Corp LLC) for analysis. The data was described using median, interquartile range, maximum and minimum. Frequencies and percentages were used in describing the categorical data.

## Results

Table 1 demonstrates the socio-demographic characteristics of 116 study participants. The median age of the participants was 37 years (IQR 22–65) with majority (33.6%) aged between 38–47 years. Approximately 35.3% of participants were male and 64.7% were female. About 40.5% of study participants did not receive formal education and majority (58.6%) were married. Many (51.7%) of the study participants were engaged in skilled occupations and few (10.3%) consumed alcohol.

**Table 1:** Socio-demographic characteristics of study participants (N = 116).

Variable	Number (n)	Percentage (%)
**Age (years)**		
Median		37 (22,65)*
18–27	22	19.0
28–37	37	31.9
38–47	39	33.6
>48	18	15.5
**Sex**		
Male	41	35.3
Female	75	64.7
**Educational level**		
No formal education	47	40.5
Prim/JHS/SHS	42	36.2
Tertiary	11	9.5
Vocational	16	13.8
**Marital Status**		
Married	68	58.6
Single	34	29.3
Cohabiting	6	5.2
Widow/widower	8	6.9
**Occupation**		
Employed	19	16.4
Unemployed	37	31.9
Self employed	60	51.7
**Religious status**		
Christianity	93	80.2
Muslim	20	17.2
Others	3	2.6
**Alcohol intake**		
Yes	12	10.3
No	104	89.7
**Do you know your sexual partner’s HBV status?**		
Yes	23	19.8
No	93	80.2

*Interquartile range (IQR).

**Fig 1:**
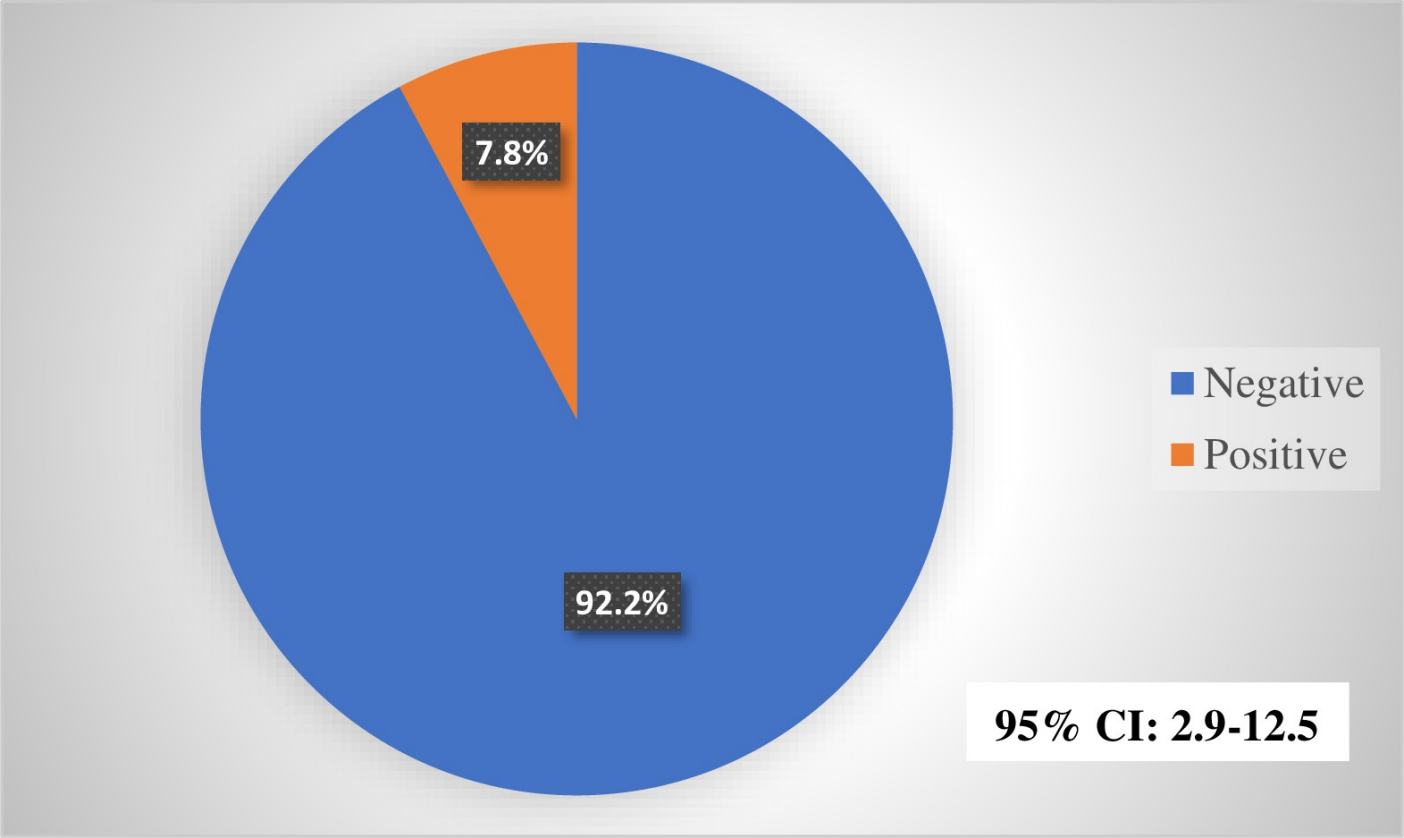
Prevalence of HBsAg among study participants (n = 116).

Table 2 and Fig 1 present serologic data of the 116 HIV study participants. After screening for HBV seromarkers, 7.8% (n = 9), 12.1% (n = 14) and 25.9% (n = 30) were solely positive for HBsAg, HBsAb and HBcAb respectively. Of the number positive solely for HBcAb, 9 (30.0%) reported a history of HBV vaccination and 21 (70.0%) did not. Nine (9) participants (7.8%) were positive for both HBcAb and HBsAg. Of those who were solely HBcAb positive, 16.7% (5/30) had detectable HBV DNA.

**Table 2:** HBV serological and molecular markers of study participants.

Serological Marker	Study Participants	
	Total (N = 116)	
	n	%
HBsAg alone	9	7.8
HBsAb alone	14	12.1
HBcAb alone^a^	30	25.9
HBcAb + HBsAg	9	7.8
HBcAb + HBeAg	6	5.2
HBcAb + HBsAb	0	0.0
HBcAb + HBeAb	3	2.6
HBcAb with DNA^b^	5/107	4.7
HBcAb without DNA^c^	102/107	95.3

^a^Hepatitis B core antibody only positive with all other markers testing negative.

^b^Hepatitis B core antibody positive with HBV DNA detected after PCR on all samples excluding HBsAg positives.

^c^Hepatitis B core antibody positive with no HBV DNA detected after PCR on all samples excluding HBsAg positives.

**Fig 2:**
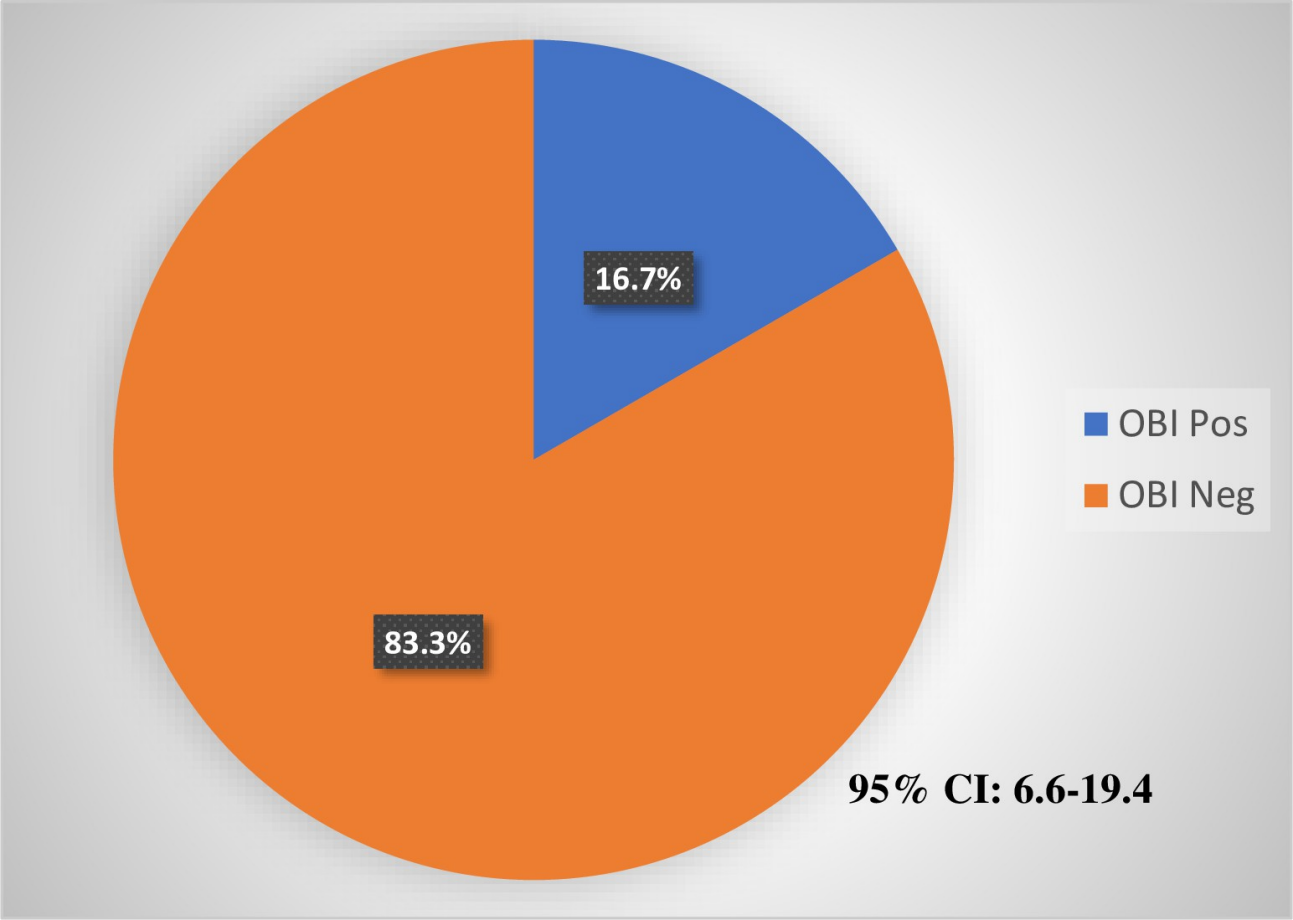
OBI prevalence among study participants positive for only HBcAb (n = 30).

**Fig 3:**
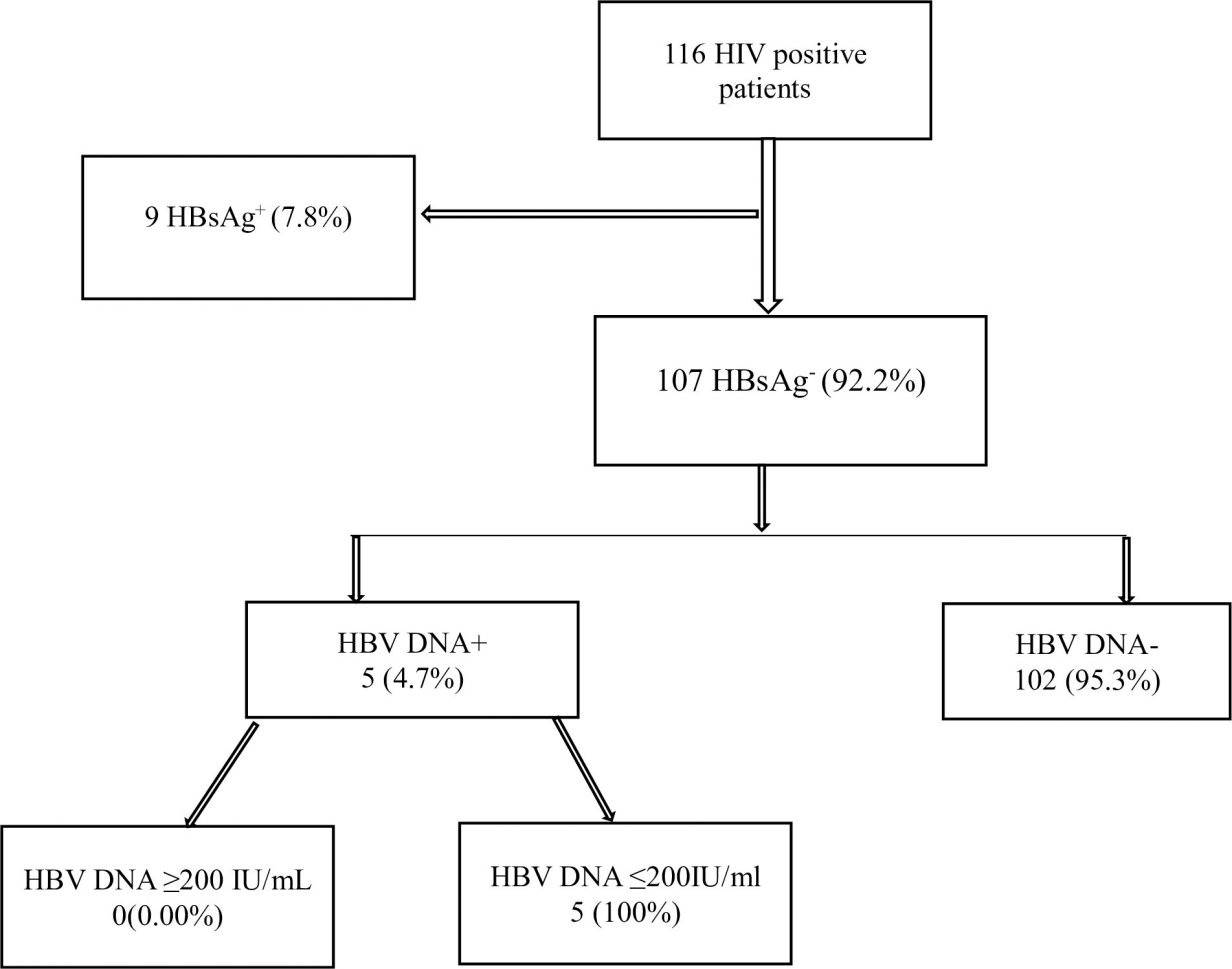
Summary of the study findings.

Table 3 and Fig 2 provides an overview of Occult Hepatitis B Virus Infection (OBI) and its associated viral load distribution among the study participants. Of the 107 participants who were HBsAg negative, 4.7% (5/107) were identified as having OBI. The median HBV DNA (viral load) for participants with OBI was 139.2IU/ml with an IQR of 96.7–142.0. All cases demonstrated viral copies less than 200 IU/ml. Fig 3 summarizes all the findings from the laboratory analysis.

**Table 3:** OBI prevalence and viral load distribution.

HBsAg negative (N = 107)	n	%
HBV DNA Positive	5	4.7
Negative	102	95.3
**Viral load for OBI**		
Median		139.2 (96.7,142)*
≤200 IU/ml	5	100
≥200 IU/ ml	0	0.0
*Interquartile range (IQR)		

## Discussion

Having hepatitis B infection alongside HIV can be a dual burden, complicating both conditions and frequently resulting in more adverse outcomes [23]. OBI presents an extra hurdle to PLHIV [24] as it is defined by HBV viral loads less than 200IU/mL (true OBI) and or above 200IU/mL (false OBI) in individuals negative for both HbsAg/HBcAb or negative for HBsAg but positive for HBcAb. In Ghana, the potential consequences of OBI in individuals with HIV remain uncertain from a clinical standpoint. Hence, the study aimed at detecting OBI among PLHIV using molecular testing. OBI in PLHIV is important to consider because the choice of ART regimen among PLHIV is influenced by the presence of HBV co-infection, with Tenofovir in addition to a second antiviral agent known to have activity against both viruses being the recommendation regimen for achieving global targets towards hepatitis B elimination in PLHIV [25]. In Ghana, tenofovir-emtricitabine/lamivudine combination is adopted as a first line option for HIV-HBV coinfected persons [26].

In this study, the overall prevalence of OBI among patients who were HBsAg negative (n = 107) was 4.7%. Among participants positive for only HBcAb (n = 30) the proportion with detectable HBV DNA was 16.7%, and the HBV DNA level was less than 200 IU/mL in all cases. In most cases of OBI, the infection is by a replication competent HBV, however in few cases the low HBV viral load indicates a suppression of viral replication and gene expression as a result of a mutation in the S or the Pol genes [27,28]. Also, the negative HBsAg could be associated with S escape mutants that are not detectable by routine serologic diagnosis [27].

There is limited data available on the prevalence of OBI among PLHIV in Ghana. In a study by Attiku et al among PLHIV in Ghana in 2021, 30.8% (n = 13) of participants who were HBsAg negative had detectable HBV DNA. However, in their study, PLHIV were on treatment for HBV [29] Similarly, a study by Dzudzor et. al., among chronic kidney patients on hemodialysis in Ghana in 2024 who were HBeAg negative but HBcAb positive, found 7.3% had detectable HBV DNA [30] The OBI prevalence found in this study was relatively lower compared to a study in South Africa (19.1%) [31–33], and significantly lower than another study among PLHIV in Morocco (68.4%) [34]. Nonetheless, a relatively lower prevalence has been reported elsewhere. The overall burden of OBI in Africa is 11.2% [33] and in West Africa, OBI prevalence varies from 15% in Ivory Coast [35] to 6.9% in Cameroon [36] among PLHIV. The difference in prevalence of OBI could be attributed to factors such as the endemicity of HBV in a particular country, HIV prevalence and very importantly, HBV screening protocols.

In this study, 39 PLHIVs (33.6%) tested positive for HBcAb, which suggests prior exposure to HBV. Among them, 9 (23.1%) tested positive for HBsAg. When compared to the previously reported prevalence rate of 41.6% for HBcAb among HIV patients in Egypt by Abdelhaziz [13], our study demonstrates a lower HBcAb positivity rate but higher when compared to a study in Nigeria where the rate was 28.0% [37].

The study outcomes have public health implications such as spread of HBV infection via vertical transmission by women of reproductive age living with HIV [38,39]. The absence of a universal birth dose vaccination program for hepatitis B in Ghana and the limited availability of affordable hepatitis B immunoglobulin pose significant concerns for the potential of vertical transmission among women living with HIV who may screen negative during routine HBsAg testing at antenatal clinic if they have OBI.

The study found the prevalence of HbsAg among study participants to be 7.8%. The HbsAg prevalence in this study is lower compared to the national hepatitis B prevalence of 8.5% [40]. Also, in comparison with the rate of HBsAg among PLHIV, the prevalence of HBsAg recorded in this study differs from previously reported rate of 6.1% [40] and 13% in a study elsewhere in Ghana [39]. The variation in prevalence could be due to differences in testing methods employed or the quality of test kits in each study and regional variations in HBV burden within the country [41].

This study also found that 12.1% of study participants were positive for HBsAb. This is however lower compared to 35.5% and 17.8% that were reported in Egypt respectively [13]. Ghana has had an Expanded program for immunization which includes HBV vaccination at 6,10, and 14 weeks for infants since 2002. Hence, the difference in anti-HBs prevalence in the two countries could be as a result of the difference in national HBV vaccination programs or attributed to variation in HBV endemicity.

Though the use of NAT in the diagnosis of OBI is the gold standard, the use of HBV serological profile test kits may help bridge the gap of inaccessible or expensive HBV DNA testing using NAT among the general populace in Ghana, especially among HIV positive individuals. The findings from this study may help policy makers in the Ghana Health Service, and especially the National AIDS Control Programme to take interest in implementing policies or protocols that could help in identifying OBI among HIV positive individuals to ensure comprehensive management of the PLHIV based on their HBV status.

## Conclusion

This study on the prevalence of OBI among PLHIV in Cape Coast demonstrates a potentially more comprehensive picture of the HBV rate among PLHIV in our setting. Testing for OBI in PLHIV may identify additional cases of HIV/HBV co-infection compared with using traditional serological tests alone and may help inform treatment choices to reduce the incidence of complications.

## Supporting information

S1 File OBI data final. (XLSX)

S2 File OBI qPCR data. (XLSX)
